# Editorial: Coding Properties in Invertebrate Sensory Systems

**DOI:** 10.3389/fphys.2016.00688

**Published:** 2017-01-10

**Authors:** Anders Garm, Berthold G. Hedwig, Sylvia Anton

**Affiliations:** ^1^Marine Biological Section, Department of Biology, University of CopenhagenCopenhagen, Denmark; ^2^Department of Zoology, University of CambridgeCambridge, UK; ^3^Neuroéthologie-RCIM, Institut National de la Recherche Agronomique-Université d'AngersBeaucouzé, France

**Keywords:** sensory signal extraction, temporal coding, neuro-ethology, olfaction, vision, mechanoreception, heat detection

Animals adapt their behavior according to the environment and their specific needs in a given situation. In order to do so in an appropriate way, they need to detect, analyze, and code the relevant sensory cues. This task is handled by sensory systems and their associated parts in the central nervous system. With few exceptions, the amount of information present in the environment and thus in principle available to sensory systems, is close to infinite. It is impossible and not desirable to encode and process all the information. Therefore, the first and most important task of any sensory system is to filter and select only the essential information—information, which potentially will improve the fitness of the bearer. Differences in sensory information processing occur between animals of different organization levels. Sensory coding in invertebrates and vertebrates relies on multiple stages of processing to extract information relevant to the survival of the individual. The wide range of organizational complexity, in combination with their relatively simple and accessible nervous systems, makes invertebrates excellent models to study general sensory coding principles. In addition, many invertebrate species are of socio-economic importance as pollinators, crop pests, or as disease vectors or elicitors. Therefore, understanding their communication systems and sensory biology is important for the development of insect management or plant protection strategies.

In the present Research Topic in Frontiers in Invertebrate Physiology we present a series of original papers and reviews illustrating the current directions of this field. The different contributions indicate not only common features of sensory coding across invertebrate phyla, but also similar processing features of complex stimuli between different sensory modalities. This is interesting, because the characteristics of the different types of sensory stimuli are inherently different, and require different types of detectors and potentially different ways of integration in nervous systems. The majority of the papers treat the coding of olfactory information with its multidimensionality, which was supposed to function under different operational constraints than other sensory modalities, and has been a field of high research activity over the past years. However, the articles of this Research Topic show that the extraction of behaviourally relevant signals from all incoming environmental stimuli, as well as the detection of low intensity signals and the analysis of temporal features can be similar across different sensory modalities, including olfaction, vision, mechanosensation, and heat perception.

The papers treating the coding of olfactory information include work on temporal processing and signal extraction in complex environments, and its behavioral outcomes as a function of physiological state, as well as potential applications of the findings to control harmful insects.

One of the major challenges for olfactory systems is to extract behaviourally relevant information from highly complex and dynamic signals. Hellwig and Tichy propose in a perspective article that olfactory receptor neurons in cockroaches, which signal sudden changes in the concentration of olfactory stimuli as compared to a constant background stimulation, might be used for tracking behaviourally relevant odor plumes. The fact that OFF neurons code better for falling concentration changes than ON neurons suggests, that they play a role in alerting a loss of an odor plume. The paper by Brill et al. shows that parallel processing of olfactory information via two antennal lobe output tracts in the honey bee comprises coincident activation patterns of projection neurons within and across parallel tracts. The results from simultaneous recordings of olfactory projection neurons in both tracts support the role of spike timing in coding olfactory information (temporal code). Another physiological property of the insect antennal lobe, which is potentially important in temporal coding of insect olfaction, is the afterhyperpolarization-phase of the projection neurons. Lei et al. show in their paper, through pharmacological experiments and modeling, some of the control mechanisms of the afterhyperpolarization phase, and confirm the involvement in temporally resetting the system for further odor-specific responses.

Another challenge for olfactory systems is the extraction of a highly relevant signal from an odor background, such as detection of moth sex pheromones in a rich background of plant volatiles. Whereas the common belief was that sex pheromone and plant odors are processed and encoded by two distinct pathways in the insect brain, Rouyar et al. show for the first time that a structurally dissimilar plant-derived odorant is able to activate the pheromone specific pathway and thus might influence pheromone processing. Badeke et al. demonstrate, however, that even though high concentrations of single plant odors can influence female tracking in the male noctuid moth *Heliothis virescens*, sex pheromone-guided behavior is not influenced by plant odors at natural concentrations.

Sensory processing and integration of different stimuli in higher brain centers ultimately leads to adequate motor output. The review by Namiki and Kanzaki addresses the function of the lateral accessory lobe in the insect brain, which is at the interface between multimodal sensory input and locomotor output. This brain area is believed to be an important output region of the brain for the control of locomotion. In a comparative approach, structure and function of lateral accessory lobe neurons in different insect species are discussed.

Because olfaction is widely used by many insect species, it has become also an important model for sensory ecology and is exploited to develop alternative methods to control harmful insects. Reisenman and Riffell review the neurobiology of host plant selection in the moth *Manduca sexta* in an ecological context. Reisenman et al. evaluate how results from the field of neuroethology can be used to elucidate how harmful insects, be it crop pests or insects transmitting diseases, trace the odors of their host plants or animal hosts. They summarize the current knowledge on the use of semiochemicals, and how results from applied studies improve our knowledge on detection and processing of olfactory signals.

The tracking of host plants sometimes changes between different life stages, and Wu et al. show that one of the odorant binding proteins (OBPs), binding to attractant semiochemicals, is upregulated in mated females of a pest fruit fly. This upregulation possibly accounts for an increased attraction to their host plant. The role of OBPs in chemosensory detection and coding is also addressed by Sun et al. Although OBPs are often found to be necessary for odor discrimination, the authors show that the function of homologous OBPs in bugs can change between species to binding of non-volatiles in the gustatory system.

The visual system basically counts the number of photons from a certain direction during a defined time period, and potentially also measures their energy (color) and their polarity. The challenges here are to extract behaviourally relevant information under limiting environmental conditions. Two papers of this Research Topic deal with coding in the visual system under low light conditions. Two strategies to enhance vision in darkness are through temporal and/or spatial summation of the photons, which will increase sensitivity on the cost of temporal and spatial resolution, respectively. Nocturnal bees enhance sensitivity through optical specializations but not through temporal summation, which would probably hamper their flight control at night (Baird et al.). Garm et al. examine vision in a nocturnal box jellyfish, which enhances sensitivity by having both a low temporal and spatial resolution. This visual system seems to be optimized to code only one highly specific information, the direction of bioluminescent flashes indicating high prey densities.

Two papers in this Research Topic deal with different forms of heat perception. The molecular actors of heat perception have been very little investigated so far. Junca and Sandoz tested the association of a heat shock with aversive olfactory conditioning of the sting extension response, and show that the TRP channel HsTRPA may be involved in heat detection in honeybees. Infrared (IR) sensing has been considered as either a specialized type of vision or thermoreception. In Jewel beetles, Schneider et al. propose a theory, which would allow bimodal photomechanic sensilla housed in IR organs to increase sensitivity to weak IR signals during flight, by making use of muscular energy coupled out of the flight motor. This mechanism could explain the capacity of these beetles to detect wood fires over distances of more than 100 km, allowing to find the resources for larvae developing in fire-killed trees.

Two contributions deal with different aspects of processing and coding of mechanosensory information. Hedwig reviews how a series of filters at different levels of the auditory pathway is used to process and code the male song by the female cricket. Carrier frequency, pulse duration and the pulse pattern are serially processed and finally tune the female phonotactic behavior to the characteristic properties of the male calling song. The other paper examines touch sensing in leeches. Kretzberg et al. show that touch- and pressure-sensitive cells in the skin, converging on common interneurons, both use quantitative and temporal elements to encode the precise location of tactile stimuli in order to elicit minimal, but precise avoiding behavior.

Altogether the work presented in this Research Topic shows the advantage of studying coding of behaviourally active sensory stimuli in invertebrates of different phyla as it reveals common features in the processing of complex sensory signals and different modalities.

## Author contributions

All authors listed, have made substantial, direct and intellectual contribution to the work, and approved it for publication.

### Conflict of interest statement

The authors declare that the research was conducted in the absence of any commercial or financial relationships that could be construed as a potential conflict of interest.

